# Local synteny and codon usage contribute to asymmetric sequence divergence of *Saccharomyces cerevisiae *gene duplicates

**DOI:** 10.1186/1471-2148-11-279

**Published:** 2011-09-28

**Authors:** Lijing Bu, Ulfar Bergthorsson, Vaishali Katju

**Affiliations:** 1 Department of Biology, University of New Mexico, Albuquerque, NM 87131, USA

## Abstract

**Background:**

Duplicated genes frequently experience asymmetric rates of sequence evolution. Relaxed selective constraints and positive selection have both been invoked to explain the observation that one paralog within a gene-duplicate pair exhibits an accelerated rate of sequence evolution. In the majority of studies where asymmetric divergence has been established, there is no indication as to which gene copy, ancestral or derived, is evolving more rapidly. In this study we investigated the effect of local synteny (gene-neighborhood conservation) and codon usage on the sequence evolution of gene duplicates in the *S. cerevisiae *genome. We further distinguish the gene duplicates into those that originated from a whole-genome duplication (WGD) event (ohnologs) versus small-scale duplications (SSD) to determine if there exist any differences in their patterns of sequence evolution.

**Results:**

For SSD pairs, the derived copy evolves faster than the ancestral copy. However, there is no relationship between rate asymmetry and synteny conservation (ancestral-like *versus *derived-like) in ohnologs. mRNA abundance and optimal codon usage as measured by the CAI is lower in the derived SSD copies relative to ancestral paralogs. Moreover, in the case of ohnologs, the faster-evolving copy has lower CAI and lowered expression.

**Conclusions:**

Together, these results suggest that relaxation of selection for codon usage and gene expression contribute to rate asymmetry in the evolution of duplicated genes and that in SSD pairs, the relaxation of selection stems from the loss of ancestral regulatory information in the derived copy.

## Background

The appearance of novel biochemical traits contributing to phenotypic diversity is inextricably linked with the constant input of new genetic fodder via gene and genome duplication. However, a mere duplication of an ancestral locus far from guarantees the origin of a novel gene product and the majority of gene duplicates end up being silenced following a brief evolutionary existence [1,2]. For those paralogs that emerge unscathed by deleterious mutations, the first clues as to how paralogs are able to forge an independent evolutionary trajectory may be provided by studying their patterns of expression divergence and relative rates of molecular evolution.

Early studies of DNA sequence divergence between paralogs suggested there was little or no difference between duplicate gene-copies in their rates of evolution [3-7]. These results were used to argue against the hypothesis proposed by Ohno that following gene duplication, one copy is under relaxed selection and begins to accumulate previously 'forbidden' mutations [2]. However, these analyses may have had limited power to detect differences in evolutionary rates, or rate asymmetry, because they analyzed old duplicates, while an increase in the evolutionary rate is easiest to detect in young gene duplicates [8]. Subsequent studies have demonstrated relatively large rate asymmetry between duplicate genes [9-13]. For instance, 20%-30% of paralogous gene in *Saccharomyces cerevisiae *displayed significant differences in evolutionary rate [11] and one or both paralog(s) exhibited accelerated evolution in 17% of the cases [12].

The phrase "gene duplication" appears to imply that all functionally relevant features of an ancestral gene are duplicated and therefore the two resulting gene copies ought to be functionally equivalent. In fact, there may be numerous differences between the two "copies". The derived copy often does not retain the full regulatory element repertoire of the ancestral copy or has some structural or genomic location differences relative to the ancestral gene [8,14-16]. These differences suggest that the derived copy might be expected to evolve under divergent constraints relative to the progenitor gene, either due to relaxation of natural selection or due to selection for novel attributes. In the majority of studies where asymmetric divergence has been established, there is no indication as to which gene copy, ancestral or derived, is evolving more rapidly. 'Derived' and 'ancestral' in the context of this study refer to the location of the paralogs in the genome rather than function. Recently, a study of gene duplicates in the mouse genome found that relocated gene copies following duplication, and in particular retrotransposed copies, evolved faster than paralogs in their ancestral location [16]. Similarly, a study in four mammalian genomes found that genes that came to reside in a different location following gene duplication were more likely to display evidence of adaptive evolution relative to gene copies that did not relocate [17].

In the case of a new gene-copy originating from a small-scale duplication (SSD) event and relocating some genomic distance from the ancestral copy, the identity of the ancestral and derived copies can be established by conservation of synteny flanking the paralogs or chromosomal location in comparison to a single-copy ortholog in an outgroup genome [15,16]. Distinguishing the ancestral from the derived copy becomes problematic in the case of whole-genome duplication (WGD henceforth). For example, in the instance of a genome resulting from allopolyploidy where duplicate gene-copies result from hybridization rather than gene duplication, naming ancestral and derived genes has no biological relevance.

Here we examine paralogs with low synonymous divergence in the *S. cerevisiae *genome to determine if it is the derived copy that evolves faster than the ancestral copy following gene duplication. Most duplicates in yeast originated from a WGD event [12,18] and for reasons mentioned in the preceding paragraph, it is inappropriate to assign ancestral and derived status to gene copies in the same manner as duplicates arising from SSD events. Gene duplicates that were previously identified as resulting from the WGD event are henceforth referred to as 'ohnologs' and were analysed separately from those resulting from SSD events to test if these two pools of duplicated genes behaved differently with respect to their rates of molecular evolution.

## Results

### Greater conservation of synteny in ohnologs

We initially commenced the analysis with 43 pairs of ohnologs and 15 SSD-derived gene duplicate pairs. These only included gene pairs that could be unambiguously assigned a single ortholog in an outgroup genome and the identification of local synteny conservation. Despite massive gene loss and genomic rearrangements in the evolutionary period subsequent to the WGD event, ohnologs have more extensive tracts of synteny relative to SSD-originated gene duplicates (Table 1). For instance, the average total upstream and downstream number of syntenic genes in the flanking regions for ohnologs versus SSD pairs is 19.87 and 4.67, respectively. Additionally, Wilcoxon signed-ranks tests revealed no significant difference in the extent of syntenic tracts in the upstream and downstream flanking regions within each population of yeast paralogs (ohnologs and SSD pairs).

**Table 1:** Averaged measures of synteny preservation for 43 pairs of ohnologs *versus *15 SSD pairs in the *S. cerevisiae *genome

*Synteny Measure*	*Ohnologs*	*SSD pairs*	*p-value*
Upstream continuous	1.41	0.47	0.0002
Downstream continuous	1.50	0.20	< 0.0001
Upstream continuous + Downstream continuous	2.91	0.67	
Upstream total	10.08	3.00	< 0.0001
Downstream total	9.79	1.67	< 0.0001
Upstream total + Downstream total	19.87	4.67	

For all measures of synteny (upstream continuous, downstream continuous, upstream total, and downstream total), the extent of synteny preservation is significantly greater in ohnologs relative to SSD pairs based on Wilcoxon tests.

### Rate of molecular evolution of ohnologs is decoupled from synteny conservation

Nine and zero of 43 ohnolog pairs displayed significant asymmetry based on Tajima's Relative Rate test (uncorrected for multiple comparisons) using DNA (Additional File 1, Table S1) and amino acid sequences (Additional File 2, Table S2), respectively. Of these nine pairs of ohnologs, the faster evolving copy was associated with less synteny conservation in seven instances. This would indicate that the rate of evolution for paralogs formed via polyploidization might be influenced by the degree of preserved synteny. However, a nonparametric rank correlation test testing for association between synteny (sum of upstream and downstream continuous synteny) and the number of unique nucleotide sites was nonsignificant (*Kendall's tau *= 0.0132; *p *= 0.91). Likewise, we found no significant association between synteny preservation and the number of unique sites at the amino acid level (*Kendall's tau *= 0.0086; *p *= 0.94).

### Derived gene copies originating from SSD events exhibit accelerated rates of molecular evolution

Seven of 15 SSD pairs showed significant asymmetry using a Tajima's Relative Rate test at the nucleotide and amino acid level, respectively (Additional File 3, Table S3 and Additional File 4, Table S4). Six of these seven SSD pairs exhibited rate asymmetry both at the nucleotide and amino acid level. In all seven instances of significant rate asymmetry between paralogs at the nucleotide level, the derived copy exhibited accelerated rates of molecular evolution. In six of the seven instances of significant rate asymmetry at the amino acid level, the derived copy was the faster-evolving paralog. A Wilcoxon signed-ranks test of all 15 SSD pairs showed that collectively, the derived copies tend to possess a greater number of unique sites, suggesting accelerated molecular evolution at the nucleotide level (*T *= -25.0; *p *= 0.024) as well as the amino acid level (*T *= -21.0; *p *= 0.029).

### CAI Results

**Figure 1:**
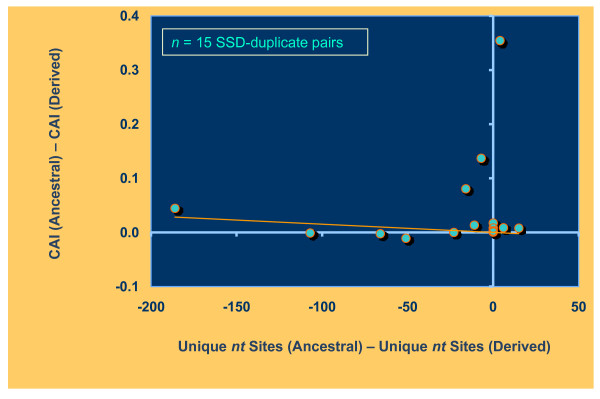
**Nucleotide sequence asymmetry and codon adaptation index (CAI) for 15 SSD pairs in the *S. cerevisiae *genome**. The sequence asymmetry measure on the *x *axis was calculated as the difference between unique nucleotide sites at the ancestral copy and the derived copy. The *y *axis represents the difference in CAI values between the ancestral copy and the derived copy for the same SSD pair. There was no significant association between differences in rate asymmetry and CAI values for SSD pairs (*Kendall's tau *= 0.226; *p *= 0.25).

**Figure 2:**
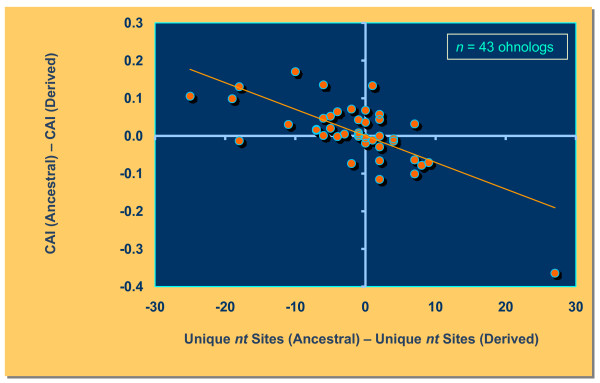
**Negative relationship between nucleotide sequence asymmetry and codon adaptation index (CAI) for 43 pairs of ohnologs in the *S. cerevisiae *genome**. The sequence asymmetry measure on the *x *axis was calculated as the difference between unique nucleotide sites at the ancestral-like copy and the derived-like copy within an ohnolog pair. The *y *axis represents the difference in CAI values between the ancestral-like copy and the derived-like copy for the same ohnolog pair. There was a significant negative correlation between differences in rate asymmetry and CIA values for ohnologs (*Kendall's tau *= -0.453; *p *< 0.0001).

Codon adaptation index (CAI) is a measure of optimal codon usage and it is positively correlated with levels of gene expression [19]. Following gene or genome duplication, there may be a period of relaxed selection resulting in lower CAI. If relaxation of selection does not apply equally to both paralogs, we may observe greater reduction in the use of optimal codons and CAI in one of the paralogs. We tested for the degree of association between the difference in CAI values between the two paralogs and the degree of rate asymmetry at the nucleotide level (difference in unique sites between the two paralogs generated from the Tajima's Relative Rate test) for both pools of gene duplicates in the *S. cerevisiae *genome. For SSD pairs, the derived paralogs have a significantly lower CAI than the ancestral paralogs (*Wilcoxon signed-ranks test*: *T *= 39.5; *p *= 0.011). However, we did not find a significant association between nucleotide rate asymmetry and change in CAI (*Kendall's tau *= 0.226; *p *= 0.25) (Figure 1). That is, faster-evolving paralogs did not have lower CAI values than slowly-evolving paralogs for SSD pairs. In contrast, we find a strong negative correlation between rate asymmetry and a difference in CAI values among ohnologs (*Kendall's tau *= -0.453; *p *< 0.0001) (Figure 2). Here, the faster-evolving paralogs resulting from the whole genome duplication event also have lower optimal codon preference.

Ohnologs and SSD duplicate pairs also differ with respect to their CAI values. The median CAI value for ohnologs and SSD pairs are 0.70 and 0.11, respectively. Indeed, CAI values averaged across both paralogs were determined to be significantly greater for ohnologs relative to SSD pairs (*Wilcoxon two-sample test*: Z = -4.723; *p *< 0.0001).

### Faster-evolving paralogs have lower mRNA abundance

The preceding CAI results suggest that relaxed selective constraints due to reduced expression of the derived paralog may contribute significantly to rate asymmetry between ancestral and derived paralogs. We find that ancestral paralogs are expressed at significantly higher levels (greater mRNA abundance) than derived paralogs for SSD pairs (*Wilcoxon signed-ranks test*: *T *= 37.5; *p *< 0.017). In contrast, ancestral-like ohnologs with greater syntenic preservation do not differ significantly in their expression levels compared to derived-like ohnologs with lower syntenic preservation (*Wilcoxon signed-ranks test*: *T *= 52; *p *= 0.54).

**Figure 3:**
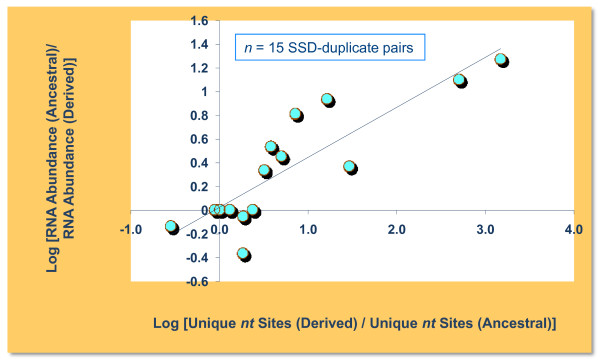
**Nucleotide sequence asymmetry and mRNA abundance for 15 SSD pairs in the *S. cerevisiae *genome**. The sequence asymmetry at the nucleotide level is expressed as the log_10_(unique sites in the derived paralog/unique sites in the ancestral paralog) and relative RNA abundance is expressed as the log_10_(RNA count for ancestral paralog/RNA count for derived paralog). There is a significant correlation between divergence between paralogs at the sequence level and divergence in their expression profiles (as represented by mRNA abundance) (*Kendall's tau *= 0.74; *p *< 0.0002).

We additionally tested if there is a relationship between transcription levels of paralogs and their degree of rate asymmetry at the nucleotide level. Figure 3 shows a significant correlation between the ratio of paralog-specific RNA and the ratio of unique sites in derived and ancestral copies of SSD pairs (*r *= 0.87, *Kendall's tau *= 0.74, *p *< 0.0002). Likewise, we find a significant association between the ratio of paralog-specific RNA and the ratio of unique sites in derived and ancestral copies for ohnologs (*r *= 0.38, *Kendall's tau *= 0.225, *p *= 0.0343).

## Discussion

Duplicated genes frequently experience an initial increase in their rate of evolution and nonsynonymous substitutions relative to synonymous substitutions. Moreover, recent analyses of young gene duplicates in several eukaryotic genomes indicate that paralogs exhibit asymmetric rates of sequence divergence in the evolutionary period soon after duplication [11,16,20-24]. Together, these observations indicate that initial relaxation of selection, or adaptive evolution, after duplication is limited to one of the paralogs, and that the slower-evolving paralog is more constrained by its ancestral function [11,22]. The majority of past studies did not distinguish between the ancestral and derived copies within a gene-duplicate pair, which in turn has precluded an unambiguous assessment of which copy is under stringent versus relaxed selective constraints.

There is some evidence that derived paralogs evolve faster than their counterparts residing at ancestral locations. In their study of evolutionarily young rodent gene duplicates, Cusack and Wolfe [16] assigned ancestral versus derived states to paralogs and demonstrated that genomic relocation of one paralog by retrotransposition engenders rate asymmetry in the sequence evolution of paralogs, commonly manifested as an accelerated rate of sequence evolution in the relocated paralog. Likewise, in bacterial genomes, the majority of paralogs that appear to have moved away from their ancestral gene neighborhood evolved faster than static paralogs [25]. Furthermore, a study of gene duplicates in four mammalian genomes determined that signatures of positive selection were more frequent in the derived copies than genes at their ancestral locations [17].

In this study, we analysed the rate of evolution in yeast paralogs for which an ancestral versus derived status could be assigned by analyzing synteny as manifested in gene-neighborhood conservation. There was significantly greater gene-neighborhood conservation in ohnologs relative to SSD pairs. Although ohnologs originated from an ancient polyploidization event and rampant genome-wide deletions have since restored functional normal ploidy in these *Saccharomyces *species [26,27], it is noteworthy that this extensive gene-neighborhood conservation has persisted. There is no difference in the extent of gene-neighborhood conservation in the upstream and downstream regions of the paralogs for both populations of duplicates (ohnologs and SSD), suggesting, on average, equal rates of preservation/loss of upstream and downstream neighboring genes.

The majority of gene duplicates with low sequence divergence in *S. cerevisiae *stem from an ancient WGD event rather than segmental duplications. Subsequent to the WGD event, there has been extensive loss of genetic material with an estimated 10% of the original ohnologs remaining [12]. Deletions of genetic material within a WGD-derived homology block have the potential to remove or rearrange regulatory sequences for the remaining genes in the block. Therefore, the DNA sequence of a paralog associated with more extensive gene-neighborhood conservation (i.e. local synteny) might be under stronger purifying selection than a paralog residing in regions that have endured more gene loss and rearrangements. While it is problematic to assign ancestral versus derived states to gene duplicates originating from WGD events, we reasoned that a paralog within an ohnolog pair could be characterized as being ancestral-like or derived-like based on the extent of gene-neighborhood conservation it shared with a single-copy ortholog in an outgroup genome. We then sought to test the hypothesis that ancestral-like gene-copies within ohnolog pairs are more likely to maintain ancestral gene function and therefore exhibit lower rates of sequence evolution. In contrast, gene-copies displaying a reduction in the extent of local synteny relative to the ortholog may be predisposed to accelerated rates of sequence evolution and the resultant fates of neofunctionalization or nonfunctionalization. However, we find no evidence of an association between rate asymmetry in ohnologs and local gene-neighborhood conservation. In other words, for ohnologs, a decline in local gene-neighborhood conservation (derived-like) does not engender accelerated rates of sequence evolution either at the nucleotide or amino acid level. This is in contrast to a study of vertebrate genomes that found a significant correlation between synteny preservation and sequence conservation [28]. We speculate that the greater number of regulatory sites in vertebrate genomes might engender greater sensitivity to syntenic changes relative to yeast. However, ohnologs in yeast do exhibit a strong significant relationship between rate asymmetry and CAI such that the faster-evolving paralogs have lower CAI. The rate asymmetry in ohnologs also seems to be to some degree caused by relaxation of selection for codon usage in one copy.

Among the SSD pairs in our sample, it is the derived copy that evolves faster on average, both at the nucleotide and the amino acid level. This lends credence to Ohno's original hypothesis that duplication enables redundancy, enabling one copy to explore new evolutionary space by accumulating mutations [2]. It is likely that segmental duplications frequently do not capture the full repertoire of regulatory sequences [8] associated with the ancestral genes and/or result in the insertion of the derived copy into a region of the genome with different chromatin structure and potentially under the influence of different regulatory elements. Under these conditions, mutations that interfere with the ancestral gene's original function would still be selected against, whereas the derived copy could be under relaxed or positive selection. For SSD pairs, the rate asymmetry at the nucleotide level is likely due to a regime of relaxed selective constraints as there is a significant reduction in the CAI of the derived paralogs within SSD pairs. The CAI compares the codon usage of a gene to codon usage in highly expressed genes; hence, the reduction in the CAI values of derived paralogs suggests that selection for optimal codon usage has been relaxed in the derived copy. Puzzlingly, we failed to detect any correlation between nucleotide sequence asymmetry of SSD paralogs and changes in their CAI values. This may stem from limited power given the small sample size of available SSD duplicates in the yeast genome.

If the rate asymmetry in paralogs is largely a consequence of relaxation of selection in the derived paralog, it should also be manifested as different levels of expression among the two copies. Previous work has shown that the evolutionary rate in yeast is strongly influenced by gene expression [29,30]. In both the yeast ohnologs and SSD pairs studied here, mRNA abundance is correlated with the rate of evolution. Moreover, within SSD pairs, it is the derived paralogs that have lowered mRNA abundance relative to the ancestral loci. Both the CAI and mRNA abundance suggest that selective constraints on gene expression is a significant driver of evolutionary rate asymmetry in paralogs.

## Conclusions

Following gene duplication, there is a general increase in the rate of evolution, and this increase is frequently asymmetric in that one paralog evolves at an accelerated pace. Asymmetry in the rate of molecular evolution after duplication has been variously associated with the evolution of novel functions, change in the number of interactions, and relaxation of selection. Here we address the related question if certain factors predispose one paralog to evolve faster. For instance, segmental duplications may translocate the derived copy to a different regulatory environment where it may evolve under different or reduced constraints [8]. Despite a limited sample of gene-duplicate pairs originating from recent small-scale duplications in *S. cerevisiae*, we find that the derived copy tends to evolve faster and is under reduced selection for codon usage. Accelerated rates in ohnologs are also associated with reduced selection for codon usage. Moreover, the rate of evolution is negatively correlated with mRNA abundance for ohnologs as well as SSD pairs. This adds to the evidence from mammals [17] that genes are not born equal and that the duplication process predisposes the derived copy to an evolutionary trajectory of initially reduced selective constraints and one that is perhaps more conducive to the evolution of new functions.

## Methods

### Identification of Gene Duplicates in S. cerevisiae with Low Synonymous Divergence

We initially selected gene families in the *S. cerevisiae *genome identified in a preceding study [31] that comprised only two members and synonymous divergence (K*_S_*) ≤ 0.35. This set had been extracted via the Genome History program [32] using the following parameters: (i) minimum translated ORF length of 100 aa, (ii) minimum number of aligned residues to accept pair being 100 aa, and (iii) using the BLAST matrix BLOSUM62 and acceptance of all BLAST hits with e ≤ 1e-07. The majority of gene duplicates within this initial sample were identified as '*ohnologs*' [33] or duplicates originating from a WGD event [12,34-37]. To further increase representation of gene duplicate pairs originating from small-scale duplication (SSD) events, we raised the K*_S _*cut-off to 1.0 for two-member families and additionally included three-member gene families with K*_S _*cut-off equal to 0.35. Ohnologs and SSD pairs in *S. cerevisiae *were distinguished by consulting Byrne and Wolfe's reconciled ohnolog list from recent comparative genomics studies [36]. The initial dataset after this first set of filtering procedures comprised 47 ohnologs and 31 SSD pairs.

### Determination of the extent of synteny preservation with outgroup genomes

Synteny blocks (regions of conserved gene order) were retrieved on the YGOB database http://wolfe.gen.tcd.ie/ygob/). For ohnologs, the single-copy ortholog within the reconstructed ancestor chromosome that is hypothesized to exist immediately before the occurrence of the WGD event 100-200 mya [37] was used as a reference outgroup. For SSD-originating paralogs, the sequence of the most recent ancestor of the paralogs was inferred based on related genes in seven post-WGD yeast species (*Saccharomyces paradoxus, S. mikatae, S. kudriavzevii, S. bayanus, S. castellii, Candida glabrata*, and *Kluyveromyces polyspora*) using the codeml program of PAML by the setting the RateAncestor = 1 [38-40]. Tajima's Relative Rate test was then performed using DNA and protein sequences in triplets containing the two focal *S. cerevisiae *paralogs and their inferred ancestral sequence. In addition, duplications involving more than one gene locus, also referred to as 'linked sets' [31] were treated as a single duplication.

We used two measures to quantify the extent of gene-neighborhood conservation of each *S. cerevisiae *paralog in its upstream and downstream flanking regions. The first measure tallied the number of continuously shared genes with the outgroup genome in both the upstream and downstream directions. The second measure tallied the total number of genes shared with the outgroup genome within a block comprising 20 loci in both the upstream and downstream flanking regions. After excluding duplicate pairs with neither synteny nor outgroup information, the sample size of our study comprised 43 and 15 pairs of ohnologs and SSD-originated duplicates, respectively (Additional Files 1-4, Tables S1-S4).

### Determining the degree of asymmetry among paralogs

Tajima's Relative Rate test [41], as implemented in MEGA version 4.0 [42] was used to determine if one of the paralogs was evolving faster. For SSD pairs, the designated outgroup sequence was a single-copy ortholog in an outgroup genome closely-related to *S. cerevisiae*. In the event that multiple outgroup species possessed a single-copy ortholog corresponding to *S. cerevisae*'s paralogs, we selected as outgroup the ortholog in the most closely-related outgroup genome. With respect to three-member gene families, the Tajima's test was only performed for the two most closely-related gene copies. For ohnologs, the outgroup was the phylogenetically closest species that contained a single-copy ortholog to the *S. cerevisiae *duplicate pair and diverged from the *Saccharomyces sensu stricto *group prior to the WGD event.

Genome and protein sequences of 11 fully sequenced yeast species were downloaded from the YGOB http://wolfe.gen.tcd.ie/ygob/ and KEGG http://www.genome.jp/kegg/catalog/org_list.html databases. Outgroup identification was performed using DNA and protein sequences of the paralogs as queries in BLASTN and BLASTP searches against the genomic and protein sequences of the 11 yeast species. The BLAST outputs were filtered and organized using a Perl script. Gene duplicate pairs and their associated outgroup sequences were first aligned with ClustalW 2.0 and then manually checked and improved, when necessary, before the analysis.

The Wilcoxon signed-rank test was used to test if, collectively speaking, the ancestral and derived copies of a gene duplicate pair are evolving at the same rate. Since the ohnolog copies could not be classified as ancestral or derived, this tests if the rate of evolution is associated with the conservation of flanking synteny. Five pairs of ohnologs with equal number of unique sites were excluded from the Wilcoxon signed-rank test to yield a final sample of 38 ohnolog pairs. For SSD pairs, the paralog with the greater upstream synteny compared to the outgroup is taken to be the ancestral copy. In the event that both paralogs have equal continuous synteny, the total synteny gene number within 20 gene loci was further included as a measure of synteny conservation. If the information above was insufficient for distinguishing the ancestral and the derived copies, the total synteny within 20 upstream and downstream gene loci was utilized.

### Relationship between codon usage, mRNA abundance and rate asymmetry

The Codon Adaptation Index (CAI) was calculated using the JCat tool http://www.jcat.de[19,43]. The JCat tool uses the method of Carbone and colleagues [44] to select a set of reference genes with optimal codon usage. In order to determine if differences in the rates of evolution are related to changes in optimal codon usage, we tested for correlation between the difference in number of unique sites (number of unique sites at the ancestral locus - number of unique sites at the derived locus) and the difference in CAI between paralogs (CAI of ancestral locus - CAI of derived locus).

An association between CAI and rate asymmetry between paralogs would suggest that gene expression is imposing differential constraints on the paralogs. As a proxy for gene expression, we obtained mRNA abundance data for all the paralogs in this study from a dataset consisting of transcript counts using single-molecule sequencing [45]. This data was used to test for an association between mRNA abundance and nucleotide rate asymmetry for both SSD pairs (Figure 3) and ohnologs.

## Authors' contributions

LB was responsible for the data collection. VK and UB conceived the study. All authors conducted statistical analyses and participated in the preparation of the manuscript. All authors read and approved the final manuscript.

## Supplementary Material

Additional file 1 ***Table S1***. Tajima's Relative Rate Test for Ohnolog DNA sequences. Click here for file

Additional file 2 ***Table S2***. Tajima's Relative Rate Test for Ohnolog amino acid sequences. Click here for file

Additional file 3 ***Table S3***. Tajima's Relative Rate Test for DNA sequences of SSD pairs using a maximum-likelihood generated ancestral sequence as outgroup. Click here for file

Additional file 4 ***Table S4***. Tajima's Relative Rate Test for amino acid sequences of SSD pairs using a maximum-likelihood generated ancestral sequence as outgroup. Click here for file
