# Supplementary material for: Local synteny and codon usage contribute to asymmetric sequence divergence of Saccharomyces cerevisiae gene duplicates
Source: BMC Evol Biol. 2011 Sep 28;11:279. doi: 10.1186/1471-2148-11-279 (PMC3190396; doi:10.1186/1471-2148-11-279)
Supplement: Additional file 1 — Table S1. Tajima's Relative Rate Test for Ohnolog DNA sequences. [file 1471-2148-11-279-S1.PDF]

Table S1: Tajima's Relative Rate Test for Ohnolog DNA sequences.

	Ancestral Paralog (A)	*Derived Paralog (B)*	*Outgroup (C)*	χ^2	*p-value*	*Unique Sites*		
						**A**	**B**	**C**
1	YBL027W	YBR084C-A	kla:KLLA0E12463g	0.03	0.85746	15	16	54
2	YBL072C	YER102W	kla:KLLA0E20559g	3.60	0.05778	2	8	72
3	YBR031W	YDR012W	kla:KLLA0B07139g	0.11	0.73888	5	4	155
4	YBR048W	YDR025W	kla:KLLA0A10483g	5.76	0.01638	5	16	41
5	YDL131W	YDL182W	kla:KLLA0F05489g	5.59	0.01810	20	38	176
6	YDL191W	YDL136W	kla:KLLA0F05247g	0.33	0.56370	1	2	39
7	YDR342C	YHR092C	kla:KLLA0D13310g	1.00	0.31731	84	90	210
8	YDR447C	YML024W	kla:KLLA0B01474g	0.22	0.63735	8	10	41
9	YEL034W	YJR047C	kla:KLLA0E22286g	0.02	0.87590	21	20	37
10	YER074W	YIL069C	kla:KLLA0C07755g	0.33	0.56370	5	7	33
11	YFR031C-A	YIL018W	kla:KLLA0D16027g	1.81	0.17793	10	17	55
12	YGL031C	YGR148C	kla:KLLA0E10857g	0.50	0.47950	14	18	44
13	YGR034W	YLR344W	kla:KLLA0B05742g	13.5	0.00024	3	21	34
14	YGR118W	YPR132W	kla:KLLA0B11231g	5.40	0.02014	12	3	28
15	YGR138C	YPR156C	kla:KLLA0E03729g	0.40	0.52454	64	57	444
16	YGR192C	YJR009C	ago:AGOS_AER031C	0.93	0.33592	11	16	169
17	YHL033C	YLL045C	kla:KLLA0E00506g	0.50	0.47950	18	14	88
18	YHR066W	YDR312W	kla:KLLA0C14586g	0.64	0.42503	35	42	349
19	YHR141C	YNL162W	kla:KLLA0D07832g	0.00	1.00000	3	3	26
20	YHR203C	YJR145C	kla:KLLA0B03652g	0.07	0.79625	7	8	79
21	YKL006W	YHL001W	kla:KLLA0B13409g	0.29	0.59298	8	6	53
22	YKR059W	YJL138C	kla:KLLA0A05731g	0.20	0.65472	2	3	188
23	YLR333C	YGR027C	kla:KLLA0B06193g	2.13	0.14440	15	8	37
24	YML026C	YDR450W	kla:KLLA0B01562g	0.20	0.65472	11	9	25
25	YML063W	YLR441C	kla:KLLA0B05060g	6.12	0.01338	20	39	59
26	YML073C	YLR448W	kla:KLLA0B04686g	0.38	0.53709	19	23	67
27	YMR121C	YLR029C	kla:KLLA0F17633g	18.69	0.00002	33	6	35
28	YMR142C	YDL082W	kla:KLLA0E22099g	16.03	0.00006	7	32	50
29	YMR143W	YDL083C	kla:KLLA0E22077g	0.14	0.70546	15	13	27
30	YMR186W	YPL240C	kla:KLLA0D12958g	0.03	0.86853	74	72	255
31	YMR230W	YOR293W	kla:KLLA0B08173g	2.58	0.10829	13	6	44
32	YNL209W	YDL229W	kla:KLLA0D19041g	0.10	0.75762	22	20	189
33	YOL120C	YNL301C	kla:KLLA0A07227g	3.85	0.04986	8	18	54
34	YOL121C	YNL302C	kla:KLLA0A07194g	0.00	1.00000	10	10	39
35	YOR133W	YDR385W	kla:KLLA0E02926g	3.00	0.08326	0	3	227
36	YOR182C	YLR287C-A	kla:KLLA0C04809g	4.50	0.03389	1	7	21
37	YOR312C	YMR242C	kla:KLLA0F08657g	0.62	0.43277	15	11	47
38	YPL079W	YBR191W	kla:KLLA0E23727g	0.14	0.70546	15	13	32
39	YPL090C	YBR181C	kla:KLLA0E24090g	0.00	1.00000	3	3	73
40	YPL198W	YGL076C	kla:KLLA0D03410g	3.20	0.07364	14	6	100
41	YPL220W	YGL135W	kla:KLLA0B02002g	0.00	1.00000	0	0	69
42	YPR080W	YBR118W	kla:KLLA0B08998g	0.00	1.00000	1	1	83
43	YPR102C	YGR085C	kla:KLLA0F08261g	1.67	0.19671	5	10	48
