# Supplementary material for: Local synteny and codon usage contribute to asymmetric sequence divergence of Saccharomyces cerevisiae gene duplicates
Source: BMC Evol Biol. 2011 Sep 28;11:279. doi: 10.1186/1471-2148-11-279 (PMC3190396; doi:10.1186/1471-2148-11-279)
Supplement: Additional file 2 — Table S2. Tajima's Relative Rate Test for Ohnolog amino acid sequences. [file 1471-2148-11-279-S2.PDF]

Table S2: Tajima's Relative Rate Test for Ohnolog amino acid sequences.

	Ancestral Paralog (A)	*Derived Paralog (B)*	*Outgroup (C)*	χ^2	*p-value*	*Unique Sites*		
						**A**	**B**	**C**
1	YBL027W	YBR084C-A	kla:KLLA0E12463g	0.00	1.00000	0	0	24
2	YBL072C	YER102W	kla:KLLA0E20559g	0.00	1.00000	0	0	24
3	YBR031W	YDR012W	kla:KLLA0B07139g	0.00	1.00000	0	0	50
4	YBR048W	YDR025W	kla:KLLA0A10483g	0.00	1.00000	0	0	15
5	YDL131W	YDL182W	kla:KLLA0F05489g	2.13	0.14440	8	15	13
6	YDL191W	YDL136W	kla:KLLA0F05247g	0.00	1.00000	0	0	13
7	YDR342C	YHR092C	kla:KLLA0D13310g	1.98	0.15990	16	25	108
8	YDR447C	YML024W	kla:KLLA0B01474g	1.00	0.31730	0	1	15
9	YEL034W	YJR047C	kla:KLLA0E22286g	0.08	0.78150	7	6	11
10	YER074W	YIL069C	kla:KLLA0C07755g	0.00	1.00000	0	0	11
11	YFR031C-A	YIL018W	kla:KLLA0D16027g	0.00	1.00000	0	0	15
12	YGL031C	YGR148C	kla:KLLA0E10857g	0.00	1.00000	2	2	19
13	YGR034W	YLR344W	kla:KLLA0B05742g	1.00	0.31730	0	1	10
14	YGR118W	YPR132W	kla:KLLA0B11231g	0.00	1.00000	0	0	3
15	YGR138C	YPR156C	kla:KLLA0E03729g	0.18	0.66980	12	10	104
16	YGR192C	YJR009C	ago:AGOS_AER031C	1.60	0.20590	3	7	42
17	YHL033C	YLL045C	kla:KLLA0E00506g	1.00	0.31730	3	1	37
18	YHR066W	YDR312W	kla:KLLA0C14586g	0.82	0.36570	4	7	104
19	YHR141C	YNL162W	kla:KLLA0D07832g	0.00	1.00000	0	0	10
20	YHR203C	YJR145C	kla:KLLA0B03652g	0.00	1.00000	0	0	19
21	YKL006W	YHL001W	kla:KLLA0B13409g	1.00	0.31730	1	0	18
22	YKR059W	YJL138C	kla:KLLA0A05731g	0.00	1.00000	0	0	61
23	YLR333C	YGR027C	kla:KLLA0B06193g	1.00	0.31730	1	0	14
24	YML026C	YDR450W	kla:KLLA0B01562g	0.00	1.00000	0	0	10
25	YML063W	YLR441C	kla:KLLA0B05060g	1.29	0.25680	2	5	14
26	YML073C	YLR448W	kla:KLLA0B04686g	0.50	0.47950	5	3	27
27	YMR121C	YLR029C	kla:KLLA0F17633g	0.00	1.00000	1	1	8
28	YMR142C	YDL082W	kla:KLLA0E22099g	0.00	1.00000	0	0	23
29	YMR143W	YDL083C	kla:KLLA0E22077g	0.00	1.00000	0	0	6
30	YMR186W	YPL240C	kla:KLLA0D12958g	2.57	0.10880	4	10	65
31	YMR230W	YOR293W	kla:KLLA0B08173g	2.00	0.15730	2	0	19
32	YNL209W	YDL229W	kla:KLLA0D19041g	0.33	0.56370	2	1	54
33	YOL120C	YNL301C	kla:KLLA0A07227g	0.00	1.00000	0	0	18
34	YOL121C	YNL302C	kla:KLLA0A07194g	1.00	0.31730	0	1	17
35	YOR133W	YDR385W	kla:KLLA0E02926g	0.00	1.00000	0	0	60
36	YOR182C	YLR287C-A	kla:KLLA0C04809g	0.00	1.00000	0	0	7
37	YOR312C	YMR242C	kla:KLLA0F08657g	0.00	1.00000	0	0	15
38	YPL079W	YBR191W	kla:KLLA0E23727g	2.00	0.15730	2	0	9
39	YPL090C	YBR181C	kla:KLLA0E24090g	0.00	1.00000	0	0	29
40	YPL198W	YGL076C	kla:KLLA0D03410g	2.00	0.15730	2	0	27
41	YPL220W	YGL135W	kla:KLLA0B02002g	0.08	0.78150	0	0	19
42	YPR080W	YBR118W	kla:KLLA0B08998g	0.00	1.00000	0	0	17
43	YPR102C	YGR085C	kla:KLLA0F08261g	1.00	0.31730	0	1	16
