# Supplementary material for: Local synteny and codon usage contribute to asymmetric sequence divergence of Saccharomyces cerevisiae gene duplicates
Source: BMC Evol Biol. 2011 Sep 28;11:279. doi: 10.1186/1471-2148-11-279 (PMC3190396; doi:10.1186/1471-2148-11-279)
Supplement: Additional file 3 — Table S3. Tajima's Relative Rate Test for DNA sequences of SSD pairs using a maximum-likelihood generated ancestral sequence as outgroup. [file 1471-2148-11-279-S3.PDF]

Table S3: Tajima's Relative Rate Test for DNA sequences of SSD pairs using a maximum-likelihood generated ancestral sequence as outgroup.

	Ancestral paralog (A)	*Derived paralog (B)*	χ^2	*p-value*	***Unique Sites***		
					**A**	**B**	**C (ancestral sequence)**
1	YDL075W	YLR406C	2.88	0.0896	5	12	2
2	YDR039C	YDR038C	0.00	1.0000	0	0	15
3	YDR533C	YOR391C	6.74	0.0094	11	27	7
4	YFL009W	YER066W	49.50	0.0000	11	77	12
5	YFL058W	YNL332W	0.00	1.0000	2	2	10
6	YGL258W	YOR387C	5.76	0.0164	5	16	17
7	YHR055C	YHR053C	0.00	1.0000	0	0	8
8	YHR056C	YHR054C	0.00	1.0000	0	0	108
9	YLR044C	YLR134W	160.17	0.0000	15	201	5
10	YNL067W	YGL147C	1.00	0.3173	21	15	8
11	YOL055C	YPL258C	0.97	0.3258	124	109	82
12	YOL086C	YMR303C	97.85	0.0000	5	112	0
13	YOR388C	YPL276W_275W	19.59	0.0000	2	25	56
14	YOR389W	YPL277C_278C	28.58	0.0000	20	71	99
15	YPL279C	YOR390W	2.00	0.1573	6	2	0

Note: Cells containing two gene IDs comprise cases where the exon-intron structure of the original locus has been altered to comprise two genes.
