# Supplementary material for: Local synteny and codon usage contribute to asymmetric sequence divergence of Saccharomyces cerevisiae gene duplicates
Source: BMC Evol Biol. 2011 Sep 28;11:279. doi: 10.1186/1471-2148-11-279 (PMC3190396; doi:10.1186/1471-2148-11-279)
Supplement: Additional file 4 — Table S4. Tajima's Relative Rate Test for amino acid sequences of SSD pairs using a maximum-likelihood generated ancestral sequence as outgroup. [file 1471-2148-11-279-S4.PDF]

Table S4: Tajima's Relative Rate Test for amino acid sequences of SSD pairs using a maximum-likelihood generated ancestral sequence as outgroup.

	Ancestral paralog (A)	***Derived paralog (B)***	χ^2	***p-value***	***Unique Sites***		
					**A**	**B**	**C (ancestral sequence)**
1	YDL075W	YLR406C	1.00	0.3173	0	1	0
2	YDR039C	YDR038C	0.00	1.0000	0	0	0
3	YDR533C	YOR391C	13.00	0.0003	0	13	0
4	YFL009W	YER066W	37.00	0.0000	0	37	0
5	YFL058W	YNL332W	0.00	1.0000	0	0	0
6	YGL258W	YOR387C	3.57	0.0588	1	6	2
7	YHR055C	YHR053C	0.00	1.0000	0	0	3
8	YHR056C	YHR054C	0.00	1.0000	0	0	42
9	YLR044C	YLR134W	59.24	0.0000	2	65	0
10	YNL067W	YGL147C	0.00	1.0000	2	2	2
11	YOL055C	YPL258C	36.94	0.0000	36	25	27
12	YOL086C	YMR303C	16.67	0.0000	2	22	0
13	YOR388C	YPL276W_275W	11.00	0.0009	0	11	7
14	YOR389W	YPL277C_278C	10.31	0.0013	8	27	26
15	YPL279C	YOR390W	3.00	0.0833	3	0	0

Note: Cells containing two gene IDs comprise cases where the exon-intron structure of the original locus has been altered to comprise two genes.
